# St. Luke’s Hospital

**Published:** 1869-12

**Authors:** Jno. E. Owens

**Affiliations:** 112 Randolph St.


### Hospital Reports.

ARTICLE I.—*St. Luke's Hospital.* Under the care of Dr. JNO. E. OWENS.

TWO CASES OF TÆNIA SOLIUM.—*Case* 1: J. C.; aged 48. Came under treatment January 1, 1869. Eighteen years ago had, for the first time, observed in the fæces segments of tænia solium. The patient's digestive organs are in good condition. Six years ago he took turpentine emulsion, which caused the expulsion, according to his own statement, of thirty feet of the worm. He informs us that he "pulled and picked his nose a great deal;" that he has suffered more or less from pain in the bowels; that his appetite is good, and sometimes craving for every meal; that he often craves food between meals. Turpentine emulsion was administered; but, occasioning considerable distress in the stomach and bowels, it was discontinued. Segments are frequently voided, and from time to time pieces of variable length. Oil of male fern was substituted for the emulsion, as follows:

| | | | |
|---|---|---|---|
| January 23, at | 9 P.M., | Oil of Male Fern, | gtt. xxv |
| " 24, at | 7 A.M., | " " | gtt. xxv |
| " 24, at | 8 " | Castor Oil, | ℥j |
| " 24, at | 11 " | Evacuation, but no traces of worm. | |
| " 24, at | 9 P.M., | Oil Male Fern, | gtt. xxv |
| " 25, at | 7 A.M., | " " | gtt. xxv |
| " 25, at | 8 " | Castor Oil, | ℥jss |
| " 25, at | 9 " | Copious evacuations, containing 9 feet of worm. | |
| " 25, at | 3 P.M., | " " " | 1 ft. in segments. |
| " 25, at | 9 " | Oil Male Fern, | gtt. xxv |
| " 26, at | 7 A.M., | " " | gtt. xxv |
| " 26, at | 8 " | Castor Oil, | ℥jss |
| " 26, at | 12 M., | Copious evacuations, without any more pieces. | |

One piece had a threadlike extremity. Dr. Hunt was good enough to examine the pieces for me, but the head was not discovered. The patient was in the habit of eating uncooked pork.

*Case* 2.—Mr. R; æt. 35; native of Ireland. Under treatment for anal fistule. One year ago, last June, he noticed, for the first time, segments of tænia solium in his evacuations. Similar segments have been voided every day or two up to this date. For the last five years he has eaten very frequently of uncooked beef.

To-day, April 19, after having been ordered to abstain from the eating both of breakfast and dinner, the following treatment was instituted; At 10 A.M., pumpkin seed emulsion (pumpkin seed, ℥ ij; water, ℥ viij; sugar, a sufficient quantity); at 12 M., castor oil, ℥j. The bowels moved freely at 6½ and 7 P.M., and at 8¼ P.M. No segments were observed. At 9.30 he had another evacuation, containing three distinct worms (tænia solium?), amounting, in the aggregate, to thirty feet. One end of each tapered to a threadlike extremity. We felt certain of finding one or more heads. Our hopes, however, were not realized, even after a careful examination. The passage of the head of the tænia solium must surely be rather a rare occurrence. We have examined eight specimens, and in no case has the head been discovered. It is likewise rare to find more than one worm. In the last case three existed. Our experience inclines us to the opinion that the treatment by pumpkin seeds is superior to that by turpentine or the oil of male fern.

112 Randolph St., *Dec.* 8, 1869.

